# The expanded amelogenin polyproline region preferentially binds to apatite versus carbonate and promotes apatite crystal elongation

**DOI:** 10.3389/fphys.2014.00430

**Published:** 2014-11-11

**Authors:** Gokul Gopinathan, Tianquan Jin, Min Liu, Steve Li, Phimon Atsawasuwan, Maria-Therese Galang, Michael Allen, Xianghong Luan, Thomas G. H. Diekwisch

**Affiliations:** ^1^Oral Biology, University of Illinois at Chicago Brodie Laboratory for Craniofacial Genetics, University of Illinois at Chicago College of DentistryChicago, IL, USA; ^2^Biocytogen, One Innovation DriveWorcester, MA, USA; ^3^Department of Periodontology, Stomatological Hospital, Jilin UniversityChangchun, China; ^4^University of Illinois at Chicago Department of Orthodontics, University of Illinois at Chicago College of DentistryChicago, IL, USA; ^5^Department of Medicine, University of ChicagoChicago, IL, USA

**Keywords:** amelogenin, hydroxyapatite, calcium carbonate, polyproline repeat proteins, vertebrate evolution

## Abstract

The transition from invertebrate calcium carbonate-based calcite and aragonite exo- and endoskeletons to the calcium phosphate-based vertebrate backbones and jaws composed of microscopic hydroxyapatite crystals is one of the great revolutions in the evolution of terrestrial organisms. To identify potential factors that might have played a role in such a transition, three key domains of the vertebrate tooth enamel protein amelogenin were probed for calcium mineral/protein interactions and their ability to promote calcium phosphate and calcium carbonate crystal growth. Under calcium phosphate crystal growth conditions, only the carboxy-terminus augmented polyproline repeat peptide, but not the N-terminal peptide nor the polyproline repeat peptide alone, promoted the formation of thin and parallel crystallites resembling those of bone and initial enamel. In contrast, under calcium carbonate crystal growth conditions, all three amelogenin-derived polypeptides caused calcium carbonate to form fused crystalline conglomerates. When examined for long-term crystal growth, polyproline repeat peptides of increasing length promoted the growth of shorter calcium carbonate crystals with broader basis, contrary to the positive correlation between polyproline repeat element length and apatite mineralization published earlier. To determine whether the positive correlation between polyproline repeat element length and apatite crystal growth versus the inverse correlation between polyproline repeat length and calcium carbonate crystal growth were related to the binding affinity of the polyproline domain to either apatite or carbonate, a parallel series of calcium carbonate and calcium phosphate/apatite protein binding studies was conducted. These studies demonstrated a remarkable binding affinity between the augmented amelogenin polyproline repeat region and calcium phosphates, and almost no binding to calcium carbonates. In contrast, the amelogenin N-terminus bound to both carbonate and apatite, but preferentially to calcium carbonate. Together, these studies highlight the specific binding affinity of the augmented amelogenin polyproline repeat region to calcium phosphates versus calcium carbonate, and its unique role in the growth of thin apatite crystals as they occur in vertebrate biominerals. Our data suggest that the rise of apatite-based biominerals in vertebrates might have been facilitated by a rapid evolution of specialized polyproline repeat proteins flanked by a charged domain, resulting in apatite crystals with reduced width, increased length, and tailored biomechanical properties.

## Introduction

Biologically produced minerals are among the functionally most important and long-lasting components of living organisms: they provide strength and defense through the formation of endo–and exoskeletons and contribute the main structural components of the powerful dentate masticatory apparatus. Biominerals are also important for auditory perception (Tohno et al., 1997) and a sense of equilibrium (Pote and Ross, 1991). Pathological examples of biomineralization include calcified arteries, ectopic bone, and renal stones, to name just a few (Russell et al., 1986). Among the major mineral constituents of biominerals are carbonates, opal, ferric oxides, and magnetites, and phosphates (Lowenstam and Weiner, 1989). Biominerals are manufactured in many different fashions, through the help of organic matrix scaffolds or in bulk, and by extracellular, intercellular, or intracellular mechanisms (Lowenstam, 1981). Protein scaffold-grown biominerals have emerged as some of the most prominent tissues in modern vertebrates, including bone, dentin, and enamel.

Commonly, two types of biomineralization proteins are distinguished: Framework macromolecules such as collagen, amelogenin and chitin (Lowenstam and Weiner, 1989) are considered organic matrix scaffolds for mineral deposition, while acidic phosphoproteins such as dentin matrix protein 1 and bone sialoprotein (George et al., 1993; Stubbs et al., 1997) are thought to act as nucleators for crystal growth. Many of the major biomineralization proteins are characterized by a high percentage of disordered residues, including dentin sialophosphoprotein (96/98%), dentin matrix protein 1 (96/96%), bone sialoprotein (83/87%), osteopontin (93/91%), amelogenin (65/70%), ameloblastin (75/88%), and enamelin (92/93%) (Kalmar et al., 2010). The tooth enamel protein amelogenin is a typical intrinsically disordered biomineralization protein as it controls the growth of apatite crystals for tooth enamel formation (Diekwisch et al., 1993; Delak et al., 2009). From a structural perspective, four distinct amelogenin domains are distinguished, the N-terminal tyrosine- and proline-rich TRAP molecule (mouse AA 1–45), the intermediary coil domain characterized by prominent histidine residues (mouse AA 46–117), the polyproline tri-peptide repeat region (mouse AA 118–164), and the glutamic acid enriched and hydrophilic C-terminus (mouse AA 165–180) (Zhang et al., 2011). A number of studies have confirmed the role of amelogenins in enamel crystal growth (Lagerström et al., 1991; Diekwisch et al., 1993; Gibson et al., 2001; Iijima and Moradian-Oldak, 2004), and the length of the amelogenin polyproline repeat region has been established as a potent regulator in the control of apatite crystal growth (Jin et al., 2009).

Apatites (Calciumhydroxyapatite, Ca_10_(PO_4_)_6_(OH)_2_) are the most common minerals in vertebrates, even though apatite minerals also occur in invertebrates, such as in the lingula shell (Iijima, 1991) and the crayfish mandible (Bentov et al., 2012). In contrast, calcium carbonates (CaCO_3_) are the major forms of biominerals in invertebrates (Lowenstam and Weiner, 1989), and are only occasionally reported in vertebrates, e.g., in otoconia (Carlström et al., 1953; Kim et al., 2011). Nevertheless, most invertebrates including the fairly evolved echinoderms feature skeletons consisting of calcium carbonate plates and spines (Gilbert and Wilt, 2011), while beginning with calcium phosphate statoliths in hagfishes and lampreys (Carlström, 1963), most vertebrates rely on calcium phosphates in form of hydroxyapatite as the inorganic building block of bones and teeth (Pasteris et al., 2008). The first evidence of a vertebrate backbone composed of hydroxyapatite dates back more than 500 million years ago to the Cambrian and Ordovician period, and vertebrate columns based on apatite biominerals became an integral component of the vertebrate body plan throughout the highly successful emergence of vertebrates until today (Benton and Harper, 2009).

In the present study we have investigated the effect of polypeptides derived from the quintessential vertebrate tooth enamel biomineralization protein amelogenin on calcium carbonate, calcium phosphate and hydroxyapatite crystal growth and mineral binding. Among the amelogenin domains studied were the N-terminal helical region, the polyproline repeat region, and the polyproline repeat region augmented by the highly charged 13 amino acid long amelogenin C-terminus. To elucidate evolutionary trends, we then traced polyproline repeat regions among eukaryotic proteins, compared the effect of the amelogenin polyproline repeat region on calcium carbonate and calcium phosphate crystal growth, and provided an explanation for differences in crystal growth through differences in mineral binding affinity.

## Materials and methods

### Proline repeat alignment

**Figure 1:**
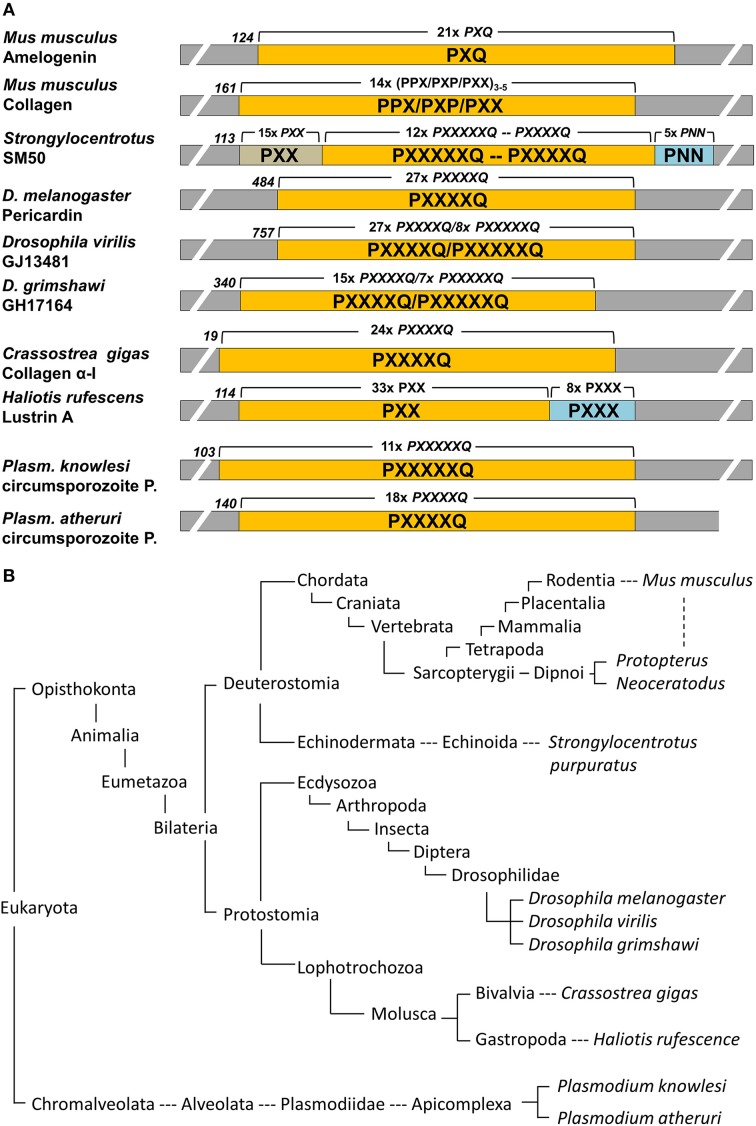
**Conservation of proline repeat elements among selected eukaryotic proteins as identified by BLAST searches. (A)** illustrates the major proline repeat element composition, the N-terminal onset of the repeat region, and the number of repeats in selected eukaryotic repeat element containing proteins. P, proline; Q, glutamine; N, Asparagine; X, any amino acid. **(B)** Corresponding eukaryotic lineages to the repeat element containing proteins listed in **(A)**, with species including the Malaria parasite *Plasmodium*, well known moluscs like the Pacific oyster and the red abalone, insects such as the fruit fly *Drosophila*, the sea urchin as a representative echinoderm, and sarcopterygians ranging from lungfishes to humans. Note that this table only lists examples and is not meant to be comprehensive.

Genbank was searched using the mouse amelogenin and SM50 polyproline repeat region as query subjects. Positive hits were further searched for repeat regions and outcomes were summarized in Figure 1.

### Proteins and peptides used in the present study

The following amelogenin polypeptides were synthesized at the UIC Protein core facility (98% purity) and used in the present study: N33, MPLPPHPGSPGYINLSYEVLTPLKWYQSMIRQP; PXX, PMQPQSPLHP MQPLAPQPPL PPLFSMQPLS PIL; PXXC, PLHPMQPLAPQPPLPPLFSMQPLSPILPELPLEAWPATDKTKREEVD; PXX12, PMQPQPPVHPMQ; PXX24, PMQPQPPVHPMQPLPPQPPLPPMF; and PXX 33, PMQPQPPVHPMQPLPPQPPLPPMFPMQPLPPML.

### Calcium carbonate and calcium phosphate nanoscale crystal growth

Calcium phosphate and calcium carbonate crystal growth experiments were modified from previous studies (Beniash et al., 2005). Briefly, peptides and proteins were dissolved in DDW at a concentration of 4 mg/ml, adjusted to pH7.5–8.0 with 20 mM NH_4_OH at 4°C and then incubated in a moisturized container with 10 mM CaCl_2_ and either 6 mM (NH_4_)_2_HPO_4_ or 6 mM (NH_4_)_2_HPO_4_ at 37°C for 2 h. Subsequently, carbon coated copper grids were immersed into the solution for 1 min and quickly rinsed with DDW, blotted against filter paper, and air dried. Transmission electron microscopy was performed using a JEOL 1220 TEM.

### Calcium carbonate macroscale crystal growth

Briefly, pH7.5–8.0 peptide/protein solutions similar to those described above (HAP Crystal Growth) were mixed with 20 mM CaCl_2_ to the final concentration of 2 mg/ml peptide/protein and 2.5 mM CaCl_2_. As a next step, 100μl of the protein/ CaCl_2_ mixture on a 12 mm cover slip deposited in a 6-well plate was transferred into a sealed container containing 200 ml of 200 mM (NH4)_2_ CO_3_. The entire container was then incubated at 37°C for 7 days, after which CaCO_3_ crystals formed on the cover slip. Cover slips were thoroughly washed with DDW to remove salt, air dried, and then subjected to Hitachi SEM for observation.

### Protein/mineral binding assay

Fourty μg of amelogenin proteins were incubated in 100 μl of calcium carbonate, calcium phosphate, or nano-hydroxyapatite solution (4μg/μl) at 37°C for 1 h. The pH of the mineral solution was adjusted to pH7.5 using glacial acetic acid or ammonium hydroxide. After 1 h incubation on a shaker, protein-HA complexes were washed with excessive amounts of 1xPBS, 3 x times at 10.000 x g to remove non-specific binding. Adsorbed protein from HA was released using 100 μl of RIPA lysis buffer. 30 μl of each sample protein were run on a 10% SDS-PAGE gel, transferred onto a PVDF membrane in a semi-dry blotting apparatus containing transfer buffer (25 mM tris-HCl, 40mM glycine, 10% methanol) for 45 min at 75 mA. The PVDF membrane was blocked with 5% bovine serum albumin for 1 h at room temperature, incubated with a chicken anti-mouse amelogenin antibody or with a rabbit polyclonal antibody directed against the N-terminal mouse amelogenin fragment at a concentration of 1:2000 for 1 h. The membrane was washed with TBST three times for 10 min each and probed with a HRP-conjugated anti-chicken secondary antibody at a concentration of 1:10,000. HRP was detected using a chemiluminescent substrate (Supersignal West Pico Chemiluminescent Substrte, Pierce).

### Crystal measurements and statistical analysis

All experiments were conducted in triplicate and crystal measurements were performed on printed electron micrographs from 10 randomly selected sample regions. Crystal dimensions per experimental group were recorded, and length and width average as well as standard deviation were calculated. Significance was determined using ANOVA and *p* < 0.05 was deemed significant.

## Results

### Polyproline repeat elements occur in a broad range of eukaryotic proteins

Polyproline repeat element proteins were identified in a wide range of eukaryotic organisms, ranging from parasites to mammals (Figure 1B). Our analysis revealed long stretches of polyproline tripeptide repeats in mammalian amelogenins and collagens, in the echinoderm spicule protein SM50, and in the moluscan shell mineralization protein Lustrin A (Figure 1A). In addition, there were polyproline hexapeptide (6mer) or septapeptide (7mer) repeats in other portions of the SM50 spicule protein, in collagen homologes of the fruitfly *Drosophila* (pericardin) and the giant oyster *Crassostrea gigas*, as well as in the circumsporozoite proteins of the Malaria parasite *Plasmodium* (Figure 1A). According to BLAST searches, there was a 47% homology between SM50 and the *Drosophila* pericardin, a 46% homology between SM50 and the *Crassostrea* collagen, and a 43–53% homology between SM50 and the *Plasmodium* circumsporozoite protein.

### In calcium phosphate crystal growth studies, the polyproline repeat region promoted the formation of thin crystallites

**Figure 2:**
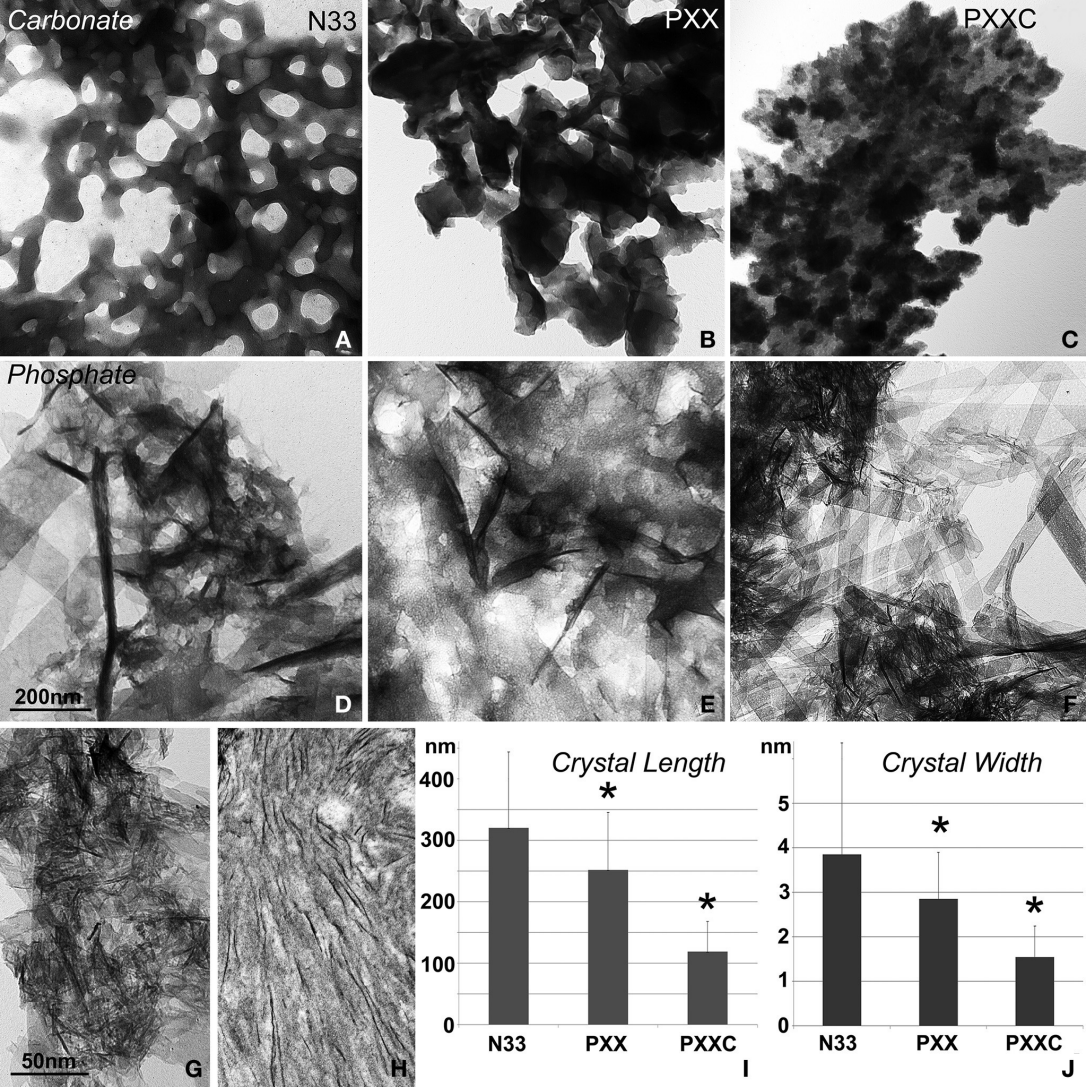
**Effect of amelogenin fragments on calcium carbonate and calcium apatite crystal growth. (A**–**C)** demonstrates the effects of three different amelogenin fragments on carbonate crystal growth, including the N-terminal 33 amino acids (N33, **A**), the PXX repeat region alone (PXX, **B**), and the PXX region augmented by the hydrophilic C-terminus (PXXC, **C**). **(D**–**F)** is similar to **(A**–**C)**, but illustrates the effects of the same amelogenin fragments on calcium phosphate crystal growth, including the N-terminal 33 amino acids (N33, **D**), the PXX repeat region alone (PXX, **E**), and the PXX region augmented by the hydrophilic C-terminus (PXXC, **F**). **(G)** is a high magnification electron micrograph of the crystal shapes generated by the augmented PXX peptide and **(H)** is a similar micrograph of early stage mouse enamel crystals of 3 days postnatal mice. The measurements in **(I,J)** correspond to figures **(D**–**F)** and compare average apatite crystal length **(I,J)** width when crystals were grown in the presence of three different amelogenin-derived peptides, including N33, PXX, and PXXC. ^*^p < 0.05.

In this set of experiments, the effects of three different portions of the amelogenin molecule on calcium carbonate and calcium phosphate crystal growth were compared (Figure 2). The N-terminal amelogenin fragment N33 caused calcium carbonate crystals to arrange in ring-shaped assemblies of hexagonal crystals, while the PXX repeat region peptide and the C-terminus augmented PXX fragment resulted in fused CaCO_3_ crystal conglomerates (Figures 2A–C). The same protein fragments resulted in entirely different crystal shapes in conjunction with calcium phosphate crystal growth environments. Here the N-terminal amelogenin N33 caused the formation of fairly large and fused crystals, while crystal dimensions were somewhat shorter and thinner when using the PXX region alone, and only the PXX repeat peptide expanded by the charged C-terminal fragment resulted in short and thin calcium phosphate crystals (Figures 2D–F,I,J). There was some similarity between the crystals generated in the PXXC calcium phosphate crystal growth experiments (Figure 2G) and natural mouse enamel apatite crystals (Figure 2H), even though the spacing between the mouse enamel crystals was wider.

### Polyproline repeat peptides with increasing length resulted in a compaction of calcium carbonate crystal assembly *in vitro*

**Figure 3:**
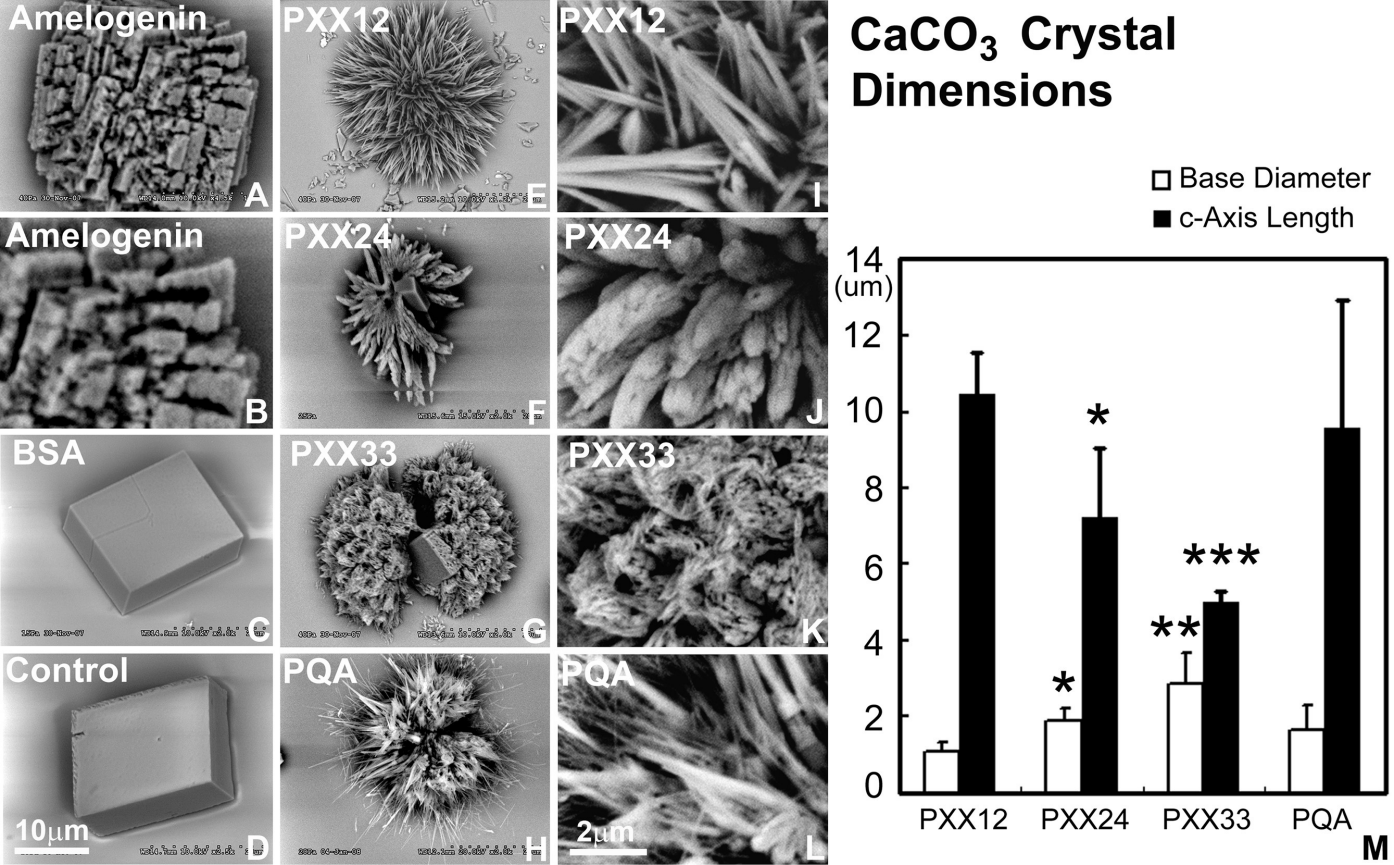
**Effect of polyproline repeat fragments of varying length on calcium carbonate crystal growth**. Scanning electron micrographs shown here document the outcomes of calcium carbonate crystal growth studies in the presence of polyproline repeat fragments of varying lengths. **(A,B)** are calcium carbonate crystals grown in the presence of amelogenin, **(C)** using bovine serum albumin as a protein control, and **(D)** is without protein. In **(E**–**G)**, polyproline repeat peptides of varying length from 12 to 33mer were used, and **(H)** is a control in which the PXX repeat was replaced by a PQA repeat. **(B,I,J,K,I)** are scanning electron micrographs recorded at five time higher magnification (magnification bars in **D** and in **I**). M illustrates the decrease in c-axis length and the increase in base diameter with increasing polyproline repeat length (from PXX12 to PXX33 and PQA as a control on the x-axis and crystal dimensions on the y-axis). ^*^p < 0.05, ^**^p < 0.01, ^***^p < 0.001.

Addition of polyproline designer peptides to the calcium carbonate crystallization solution resulted in the formation of needle-shaped calcium carbonate crystals arranged around a central core in a radial fashion (Figure 3). The PXX12 peptide yielded needles measuring 10.44 ± 1.14 μm in length and 1.10 ± 0.22 μm in thickness. The PXX24 peptides resulted in fused spicules with 7.23 ± 1.81 μm length and 1.91 ± 0.30 μm thickness. Addition of the PXX33 peptide resulted in the presence of cone-shaped processes consisting of fused individual crystallites and measuring 5.03 ± 0.27 μm in length and 2.88 ± 0.82 μm in thickness at the base. Samples without protein and samples containing albumin yielded cuboidal crystal shapes with smooth surfaces (Figure 3). Addition of amelogenin to the calcium carbonate crystallization mix resulted in cubes with rugged, deeply fissured surface structures (Figure 3). All three control conditions, no protein, BSA, and amelogenin, did not yield any needle-shaped crystal habits (Figure 3).

### The polyproline-rich amelogenin C-terminal peptides strongly bound to the calcium phosphate and hydroxyapatite mineral and not to calcium carbonate, while there was little difference in binding between the N-terminal amelogenin fragment and the three calcium minerals studied

**Figure 4:**
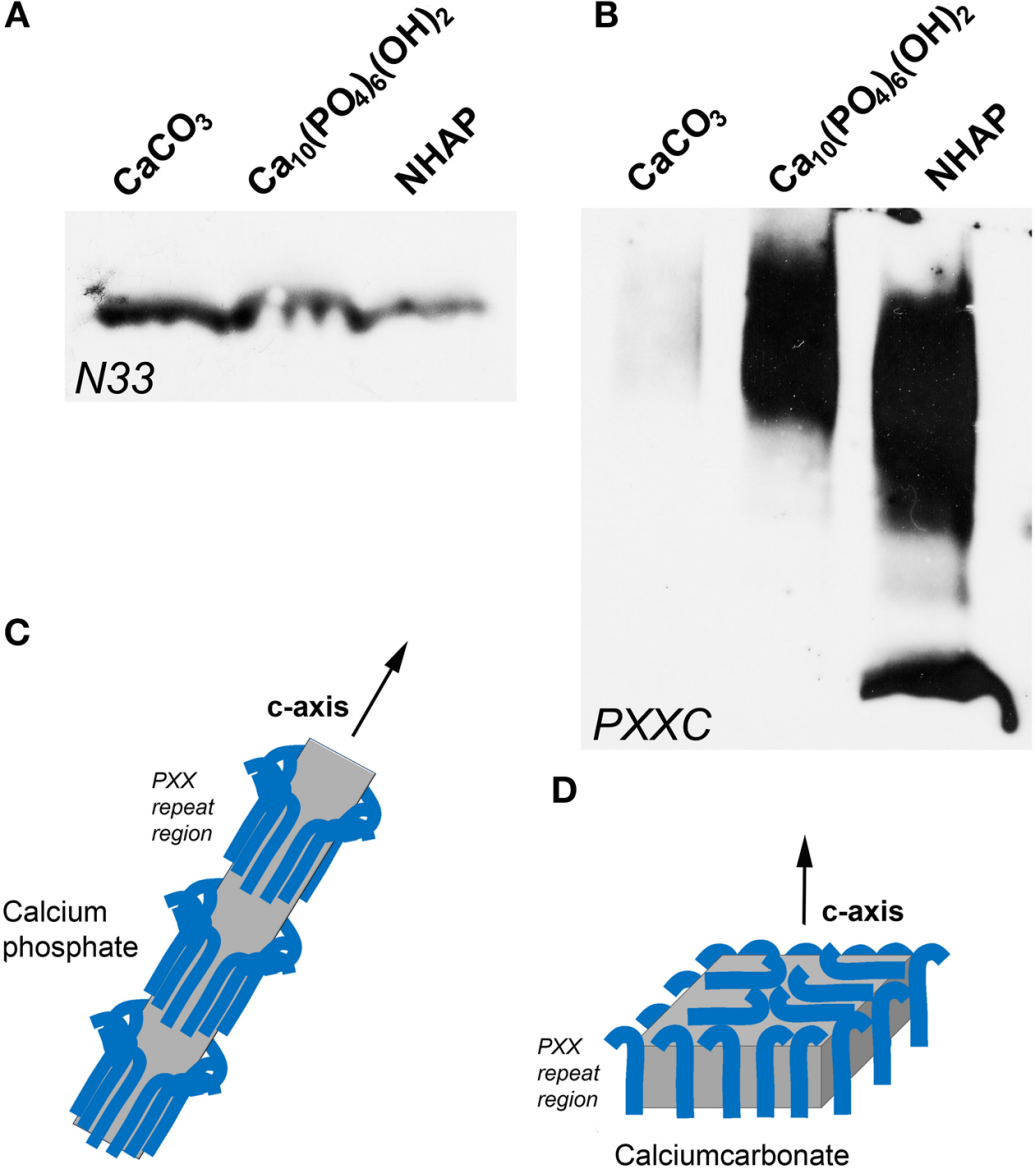
**Binding of amelogenin fragments to calcium minerals. (A,B)** illustrate results from parallel experiments in which either an amelogenin N-terminal fragment (N33, **A**) or a C-terminus augmented amelogenin repeat fragment (PXXC, **B**) were incubated with three different calcium minerals, including calcium carbonate (CaCO_3_), calcium phosphate, and nanohydroxyapatite (NHAP). **(C,D)** illustrates our explanation for the differences in calcium mineral crystal growth when subjected to different protein environments. Here we propose that polyproline repeat regions (blue hooks) as they occur in amelogenin bind to the a- and b- axis of calcium phosphate crystals and allow for the growth of long apatite crystal through expansion mostly in c-axis direction **(C)**. In contrast, proteins including polyproline peptides appear to affect calcium carbonate crystal growth equally in all directions, resulting in an overall compaction of the final crystal **(D)**.

The purpose of this experiment was to ask the question whether portions of the amelogenin molecule display preferential binding affinity to either apatite, calcium phosphate, or calcium carbonate mineral. In a first part of this experiment, the N-terminal N33 amelogenin peptide was incubated with calcium carbonate, calcium phosphate, and nanohydroxyapatite, and our binding assay revealed that N-terminal binding was approximately double as high between N33 and CaCO_3_ as it was between N33 and the two calcium phosphates employed in this study (Figure 4A). The second experiment (Figure 4B) was conducted in parallel and revealed that the C-terminus augmented polyproline repeat peptide preferentially bound to calcium phosphate and the nanohydroxyapatite minerals, while there was only faint evidence of carbonate binding. Together, this study demonstrated that the long amelogenin polyproline repeat region augmented by the charged C-terminus strongly binds to calcium phosphate and nanohydroxyapatite, while the amelogenin N-terminus preferentially binds to calcium carbonate.

## Discussion

In the present study we asked the question how portions of the highly conserved amelogenin biomineralization molecule affect calcium phosphate and calcium carbonate crystal growth and whether this effect might be explained by the interaction between amelogenin fragments and these two different calcium minerals. Amelogenin turned out to be an interesting study subject in the present inquiry because of its organization into distinct domains, its high level of conservation among individual domains, because of the presence of a distinct polyproline repeat domain, and in light of its presumed role in elongated crystal growth. Here we have tested the effect of amelogenin and some of its domains on both calcium carbonate and calcium phosphate mineral growth and binding to determine whether the interaction of amelogenin fragments with calcium phosphates when compared to calcium carbonates provided any unique insights into the evolutionary benefits of protein mediated calcium phosphate growth for vertebrate skeletal tissue function. Our study revealed that the amelogenin polyproline repeat region augmented by its hydrophilic C-terminus interacted primarily with calcium phosphates, and that this region promoted the growth of thin and individually distinct crystals, while the amelogenin N-terminus preferentially bound to calcium carbonates and inhibited C-axis crystal growth. Together, these findings provide unique insights into the protein dynamics that might have aided the evolutionary benefits of apatite-based skeletal tissues in vertebrates.

Our overview of polyproline repeat element containing proteins among eukaryotes gives testimony to the universal distribution of such proteins among eukaryotes, ranging from amelogenins to collagens, and from parasitic protists to most mammals. Some of these proteins are functionally related to biomineralization, such as amelogenins, collagens, SM50, and Lustrin A, while a group represented by the *Drosophila* pericardin proteins plays a role in heart development (Chartier et al., 2002), and others such as the circumsporozoite protein of various *Plasmodium* species are essential for the invasion of the parasite into the host organism (Plassmeyer et al., 2009). The broad distribution and multiple functions of polyproline repeat proteins suggest that the repeat region plays somewhat of a generic role that can be adapted to serve multiple functions (Adzhubei et al., 2013). However, a common element among such Polyproline-II repeat region containing proteins is their ability to form elongated fibril-type structures (Hall et al., 2014) and to interact with surfaces, either with other proteins (Adzhubei et al., 2013), or with polyphenols in saliva (Murray and Williamson, 1994), or with biological minerals such as apatites (Jin et al., 2009). These two qualities suggest that the amelogenin polyproline tri-peptide repeat region is likely engaged in interactions with the apatite surface, providing a semi-flexible elongated template for crystal growth. Our analysis also revealed that the polyproline peptide repeats were organized into either trimer, or hexamer/heptamer subunits, with tripeptide repeats as the rule in the two vertebrate proteins listed here. Subunit lengths of varying amino acid number are likely to affect the periodicity and surface exposure of critical amino acids for binding to the mineral or adjacent protein surface. Finally, the majority of the proline oligopeptide repeat regions contains a glutamine in the terminal repeat position, a feature that might be linked to the aggregation behavior of these proteins (Altschuler et al., 1997). The ability of polyglutamine proteins to aggregate is not only important for the generation of biomineralization templates (Jin et al., 2009), but also plays a role in the pathogenesis of neurodegenerative diseases, including Huntington's disease and Friedreich's ataxia (Shao and Diamond, 2007).

In our calcium phosphate crystal growth studies, the polyproline repeat region promoted the formation of thin crystallites, while the same proteins in calcium carbonate crystal growth conditions formed either ring-shaped assemblies of hexagonal crystals or fused CaCO_3_ crystal conglomerates. These findings indicate that at least under the conditions described here, the ability to form needle-shaped crystals was unique to the interaction between calcium phosphates and the three amelogenin fragments employed in the present study. The occurrence of nanoscale needle-shaped and parallel organized crystals is predominantly a feature of vertebrate biominerals such as bone, dentin, and developing enamel (Fitton-Jackson and Randall, 1956; Diekwisch et al., 1995; He and George, 2004), even though invertebrates are entirely capable of promoting needle-shaped calcium carbonate spicules, albeit with relatively thicker diameters (Beniash et al., 1999). Our data also indicated that only the C-terminus augmented polyproline fragment promoted the growth of short, thin, and parallel nanoscale crystals, while both the amelogenin N-terminus and the polyproline region alone appeared to fuse individual crystals into thicker subunits. These results suggest that the amelogenin C-terminus is necessary for the immediate protein adhesion to the calcium phosphate crystal surface (Shaw et al., 2004) and the inhibition of a- and b- axis crystal growth in favor of c-axis directed crystal elongation (Diekwisch et al., 1993). Together, this set of data indicates that the combination of apatites with the augmented amelogenin carboxy-terminus results in the growth of minerals with stereotypical vertebrate biomineralization features such as small, short, and parallel crystal assemblies, while amelogenin fragments in combination with calcium carbonates generate bulk mineral clusters and fail to produce needle-shaped crystals.

We then asked whether the elongation of the polyproline region would affect calcium carbonate crystal growth in week-long growth studies to compare the effect on crystal elongation with our previously published apatite crystal growth studies using the same peptides (Jin et al., 2009). In opposite to the apatite mineralization outcomes (Jin et al., 2009), increased PXX polypeptide repeat length resulted in shorter calcium carbonate crystals with broader basis, suggesting that PXX repeat polypeptides affected calcium carbonate crystals in a different fashion than calcium apatite crystals. We attribute these differences in crystal growth patterns to possible differences in the interaction between the polyproline repeat region and different crystal facets, with polyproline repeat regions preferentially binding to a- and b- axis facets of apatite crystals and to all surfaces of carbonate crystals. In the larger context of typical vertebrate versus invertebrate biomineralization patterns, the different effects of polyproline repeat polypeptides on axial crystal growth in apatite and carbonate crystal morphologies corresponded with naturally occurring crystal shapes, i.e., long crystals in apatite minerals enamel and bone, and compact plates in the calcium carbonate minerals calcite, aragonite, and vaterite.

Our protein/mineral binding studies revealed that the polyproline-rich amelogenin carboxy-terminal fragments strongly bound to the calcium phosphate and hydroxyapatite mineral and not to calcium carbonate, while there was little difference in binding between the N-terminal amelogenin fragment and the three calcium minerals studied. This finding is significant in the context of previous studies that emphasized the importance of the amelogenin carboxy-terminus for apatite binding (Moradian-Oldak et al., 2002; Shaw et al., 2004; Sun et al., 2008; Pugach et al., 2010). However, also other portions of the amelogenin molecule are important for enamel formation, including the N-terminus (Dunglas et al., 2002). The relationship between the C-terminus and apatite binding was early on established by Moradian-Oldak et al. (2002), demonstrating that apatite binding of amelogenins lacking the C-terminus was significantly reduced, followed by a solid state NMR study in which the charged amelogenin C-terminal region was oriented next to the hydroxyapatite surface (Shaw et al., 2004). These findings were further corroborated by enamel protease cleavage experiments resulting in reduced amelogenin-apatite binding in amelogenins of decreasing length (Sun et al., 2008) and an enamel defect mouse model that overexpressed a transgene lacking the C-terminal 13 amino acids (Pugach et al., 2010). Finally, Zhu et al. pointed to the importance of the P70 proline as part of a series of central polyproline repeats for apatite binding and self-assembly, emphasizing the role of the proline repeats in the interaction between amelogenins and apatites (Zhu et al., 2011). Here we show that the charged carboxy-terminal amelogenin motif with its adjacent polyproline repeat region preferentially binds to apatite and calcium phosphates when compared to calcium carbonate, suggesting that the C-terminal polyproline/hydrophilic stretch has evolved as part of the vertebrate biomineralization machinery in control of apatite crystal growth. This interpretation adds to previous studies reporting a trend of increasing polyproline repeat length as a means to control apatite crystal dimension during vertebrate evolution (Bonass et al., 1994; Sire et al., 2005; Diekwisch et al., 2009; Jin et al., 2009). Varying the length of polyproline repeat stretches would affect the spacing between individual crystals within supramolecular amelogenin subunit compartments (Diekwisch et al., 1993) or in between nanosphere assemblies (Jin et al., 2009).

A number of possible reasons for the predominance of hydroxyapatites in vertebrate mineralized tissues have been proposed, including (i) the abundance of calcium and phosphorus in food sources, (ii) the sparing solubility and resulting relative stability of crystalline apatites, (iii) the ability of apatites to accept chemical substitutes, including carbonate substitutes, that greatly affect their physical and biomechanical properties (Pasteris et al., 2004), and (iv) mechanical advantages of apatites over carbonates, including advantages associated with increased hardness (Reid et al., 2012). Here we have introduced another, perhaps equally important reason for the evolutionary selection of apatite as the preferred vertebrate biomineral: its ability to be readily shaped by matrix proteins through the effect of repeat region assembly and the affinity of flanking charged domains to the lateral apatite surface, allowing for c-axis crystal growth and controlled inhibition of crystal width. This ability of the organic vertebrate protein matrix to shape calcium phosphate minerals into many parallel packed, individually separated, long and thin crystallites has become the biomechanical foundation for the strong yet flexible backbones and jaws of the vertebrate skeleton, ensuring survival and success in the late Cambrian/early Ordovician biosphere and until today.

### Conflict of interest statement

The authors declare that the research was conducted in the absence of any commercial or financial relationships that could be construed as a potential conflict of interest.
